# CircARVCF Contributes to Cisplatin Resistance in Gastric Cancer by Altering miR-1205 and FGFR1

**DOI:** 10.3389/fgene.2021.767590

**Published:** 2021-11-25

**Authors:** Ruirui Zhang, Huanyu Zhao, Hongmei Yuan, Jian Wu, Haiyan Liu, Suan Sun, Zhengwei Zhang, Jiayang Wang

**Affiliations:** ^1^ Department of Pathology, Huai’an First People’s Hospital, Nanjing Medical University, Huaian, China; ^2^ Department of Pathology, Huai’an Huaiyin Hospital, Huaian, China; ^3^ Department of Rodio Chemotherapy, Huai’an First People’s Hospital, Nanjing Medical University, Huaian, China

**Keywords:** GC, DDP, resistance, circARVCF, miR-1205, FGFR1

## Abstract

**Background:** Chemoresistance is a major barrier to the treatment of human cancers. Circular RNAs (circRNAs) are implicated in drug resistance in cancers, including gastric cancer (GC). In this study, we aimed to explore the functions of circRNA Armadillo Repeat gene deleted in Velo-Cardio-Facial syndrome (circARVCF) in cisplatin (DDP) resistance in GC.

**Methods:** The expression of circARVCF, microRNA-1205 (miR-1205) and fibroblast growth factor receptor 1 (FGFR1) was detected by quantitative real-time polymerase chain reaction (qRT-PCR), western blot assay or immunohistochemistry (IHC) assay. Cell Counting Kit-8 (CCK-8) assay and colony formation assay were performed to evaluate DDP resistance and cell colony formation ability. Transwell assay was conducted to assess cell migration and invasion. Flow cytometry analysis was done to analyze cell apoptosis. Dual-luciferase reporter assay and RNA immunoprecipitation (RIP) assay were manipulated to analyze the relationships of circARVCF, miR-1205 and FGFR1. Murine xenograft model was constructed to explore DDP resistance *in vivo*.

**Results:** CircARVCF level was increased in DDP-resistant GC tissues and cells. CircARVCF silencing inhibited DDP resistance, colony formation and metastasis and induced apoptosis in DDP-resistant GC cells. CircARVCF directly interacted with miR-1205 and miR-1205 inhibition reversed circARVCF silencing-mediated effect on DDP resistance in DDP-resistant GC cells. FGFR1 served as the target gene of miR-1205. MiR-1205 overexpression restrained the resistance of DDP-resistant GC cells to DDP, but FGFR1 elevation abated the effect. In addition, circARVCF knockdown repressed DDP resistance *in vivo*.

**Conclusion:** CircARVCF enhanced DDP resistance in GC by elevating FGFR1 through sponging miR-1205.

## Highlights

1. CircARVCF is upregulated in DDP-resistant GC tissues and cells.
2. CircARVCF knockdown suppresses DDP resistance in DDP-resistant GC cells.
3. CircARVCF directly interacts with miR-1205 to regulate FGFR1 expression.
4. CircARVCF promotes DDP resistance in DDP-resistant GC cells by miR-1205/FGFR1 axis.
5. CircARVCF enhances DDP resistance of GC *in vivo.*

## Introduction

Gastric cancer (GC) is a worldwide life-threatening malignant tumor with strong a ability of metastasis and proliferation (Guggenheim and Shah, 2013; Bray et al., 2018). The patients are often diagnosed at the late stage for lacking specific symptoms and diagnostic indicators in early GC, and then lead to poor prognosis (Song et al., 2017). Surgery and adjuvant chemotherapy are the major treatment methods for GC, although molecular targeted therapy has been developed. Cisplatin (DDP) is the main first-line drug for GC treatment (Wang et al., 2019a). However, drug resistance impedes the effectiveness of chemotherapy (Galluzzi et al., 2012; Marin et al., 2016). Based on the above reasons, studying the mechanism of chemoresistance is necessary for improving the survival of GC.

Circular RNAs (circRNAs) are newly discovered non-coding RNAs (ncRNAs) that are distinguished by signal-stranded closed loop (Chen and Yang, 2015). CircRNAs are dysregulated and play vital roles in the biological and pathological processes in human cancers (Wang et al., 2017). Moreover, circRNAs can act as the competitive endogenous sponge for microRNAs (miRNAs), which then directly targeted related mRNAs to alter gene expression (Zhong et al., 2018). CircRNAs are linked to the development of chemoresistance in GC (Cui et al., 2020). For instance, circ_0000144 restrained oxaliplatin (OXA) resistance of GC *via* altering miR-502-5p and ADAM9 (Gao et al., 2021). CircFN1 promoted the malignancy and DDP resistance of GC *via* decoying miR-182-5p (Huang et al., 2020). CircARVCF (hsa_circ_0092330) was formed by the exons of ARVCF gene and was found to be upregulated in GC (Ouyang et al., 2019). However, the functions of circARVCF in the carcinogenesis and drug resistance of GC are unclear.

The involvement of miRNAs in tumor progression and chemoresistance is widely characterized (Taheri et al., 2021). For example, miR-149 facilitated 5-FU resistance in GC through binding to TREM2 (Wang et al., 2020a). MiR-127-5p impeded the resistance of GC to Apatinib (Yu et al., 2021). MiR-1205 functioned as a tumor inhibitor in diverse cancers, such as colorectal cancer (Han et al., 2021), glioma (Wang et al., 2021), ovarian cancer (Wang et al., 2020b) as well as GC (Lin et al., 2020). Even so, it remains unclear whether miR-1205 is related to the drug resistance of GC. Moreover, fibroblast growth factor receptor 1 (FGFR1) was found to contain miR-1205 binding sites, but their relationship is still unknown.

In this paper, we elucidated the functions and relationships of circARVCF, miR-1205, and FGFR1 in regulating the chemoresistance of GC, attempting to find a novel target to relieve chemoresistance in GC.

## Materials and Methods

### Tissue Collection

The GC and matched adjacent non-tumor tissues were collected from 37 GC patients (20 males and 17 females, aged 25–71) at Huai’an First People’s hospital. The enrolled patients received DDP-based chemotherapy before this surgery. According to the resistance of GC patients to DDP (Bagrodia et al., 2016), the patients were divided into two groups: chemoresistant group (*n* = 20; tumor relapse during DDP-based chemotherapy) and chemosensitive group (*n* = 17; no tumor recurrence during DDP-based therapy). All patients provided the written informed consents. The research granted approval by the Ethics Committee of Huai’an First People’s Hospital.

### Cell Culture

Human normal gastric mucosa cells (GES-1) and GC cells (N87, HGC-27, MKN-45, and AGS) were acquired from Procell (Wuhan, China). All cells were cultured in RPMI1640 (Procell) added with 1% penicillin/streptomycin (Procell) and 10% FBS (Procell) at 37°C and 5% CO_2_.

The DDP-resistant GC cells (MKN-45/DDP and AGS/DDP) were generated by continuous gradient exposing MKN-45 and AGS cells to increasing doses of DDP (Sigma-Aldrich, St. Louis, MO, United States) from 0.03 μg/ml until the cells acquired resistance to 1 μg/ml (0.05, 0.1, 0.15, 0.2, 0.3, 0.4, 0.5, 0.6 0.7, 0.8, 0.9, 1 μg/ml) for about 12 months. The DDP-resistant cells were cultured in cisplatin (1 μM)-contained RPMI1640 (Procell) and cultured in cisplatin-free RPMI1640 (Procell) for 1 week before further use.

### Quantitative Real-Time Polymerase Chain Reaction

RNA isolation was manipulated with RNAiso Plus (Takara, Dalian, China). Then M-MLV Reverse Transcriptase reagent (Promega, Madison, WI, United States) or All-in-One™ miRNA Detection reagent (GeneCopoeia, FulenGen, China) was employed on total RNA for the synthesis of cDNAs. Afterward, SYBR Green qPCR mix (Takara) was used for the reaction on an ABI 7500 Real-Time PCR system (Applied Biosystems, Foster City, CA, United States). The expression was computed by the 2^−ΔΔCt^ way. β-actin and U6 served as the internal controls. The primers were listed in Table 1.

**TABLE 1:** Primers sequences used for qRT-PCR.

Name		Primers for PCR (5′-3′)
CircARVCF	Forward	TGA​GGA​CTC​CCT​GTT​CCT​TTG
	Reverse	TTG​GTA​TGA​GGC​TGT​GAC​CG
ARVCF	Forward	CTA​TTG​TCA​CAT​CCG​AAG​ATG​GC
	Reverse	CGT​ACT​GTC​CGA​GTG​GTC​AC
miR-1205	Forward	ACA​CTC​CAG​CTG​GGT​CTG​CAG​GGT​TTG​C
	Reverse	TGGTGTCGTGGAGTCG
FGFR1	Forward	CCC​GTA​GCT​CCA​TAT​TGG​ACA
	Reverse	TTT​GCC​ATT​TTT​CAA​CCA​GCG
β-actin	Forward	ATC​AAG​ATC​ATT​GCT​CCT​CCT​GAG
	Reverse	CTG​CTT​GCT​GAT​CCA​CAT​CTG
U6	Forward	CTCGCTTCGGCAGCACA
	Reverse	AAC​GCT​TCA​CGA​ATT​TGC​GT

### Cell Counting Kit-8 Assay

To determine DDP resistance, MKN-45/DDP and AGS/DDP cells (1 × 10^4^ cells/well) were cultured in 96-well plates. On the next day, the cells were treated with varying doses of DDP (0.01, 0.1, 1, 10, and 100 μM) for 48 h. 10 μL CCK-8 (Sigma-Aldrich) was then supplemented into the well and incubated for 4 h. Finally, the absorption at 450 nm was examined by a microplate reader and the 50% inhibitory concentration (IC_50_) of DDP was analyzed.

### Actinomycin D and RNase R Treatment

For the cyclization analysis of circARVCF, MKN-45/DDP, and AGS/DDP cells were interacted with Actinomycin D (Sigma-Aldrich) for indicated times. Total RNA was managed with RNase R (3 U/μg; Epicenter, Madison, WI, United States) at 37°C for 20 min. Afterward, the levels of circARVCF and ARVCF were detected via qRT-PCR.

### Cell Transfection

Specific small interfering RNAs targeting circARVCF (si-circARVCF#1, si-circARVCF#2, and si-circARVCF#3) and scramble control si-NC, miR-1205 mimics (miR-1205), inhibitors (in-miR-1205) and related controls miR-NC and in-miR-NC, FGFR1 overexpression vector (FGFR1) and empty control pcDNA, short hairpin RNA targeting circARVCF (sh-circARVCF) and sh-NC were synthesized by GeneCopoeia (Guangzhou, China). Then MKN-45/DDP and AGS/DDP cells were seeded into 24-well plates and transfected with the oligonucleotides or vectors through Lipofectamine 3,000 (Invitrogen, Carlsbad, CA, United States) according to the manufacturers’ instructions.

### Colony Formation Assay

MKN-45/DDP and AGS/DDP cells (300 cells/well) were planted into 6-well plates and maintained for 2 weeks. The medium was changed every 3 days. The colonies were fixed with 4% paraformaldehyde (Sigma-Aldrich) and then dyed with 0.1% crystal violet solution (Sigma-Aldrich). After that, the colonies were calculated.

### Transwell Assay

By using the transwell insert chambers (Corning, Corning, NY, United States) with or without Matrigel (Corning) coverage, cell invasion and migration were assessed. In short, MKN-45/DDP and AGS/DDP cells (1 × 10^4^) were resuspended in 200 μL serum-free medium was added into the top chamber. The 600 μL complete medium was filled into the lower chamber. Following 24 h of incubation, the migrated or invaded cells were fixed with 4% paraformaldehyde (Sigma-Aldrich) anddyed with 0.1% crystal violet (Sigma-Aldrich). Next, the number of migrated or invaded cells was counted via a microscope (100×; Olympus, Tokyo, Japan) through the five random areas.

### Flow Cytometry Analysis

After indicated transfection, MKN-45/DDP and AGS/DDP cells were resuspended in binding buffer and double-dyed with Annexin V-fluorescein isothiocyanate (FITC) and propidium iodide (PI) for 15 min without light based on the instructions of the Apoptosis Detection Kit (Beyotime, Shanghai, China). The apoptotic cells were detected using flow cytometry.

### Western Blot Assay

After being extracted from tissues and cells with RIPA buffer (Beyotime), the proteins (20 μg) were separated through 10% SDS-PAGE electrophoresis. Then the proteins were transferred onto PVDF membranes, followed by blockage in 5% skim milk for 2 h. Subsequently, the membranes were maintained overnight with primary antibodies at 4°C and corresponding secondary antibody (bs-0293G-HRP; 1:10,000; Bioss, Beijing, China) for 2 h at room temperature. The protein blots were subjected to ECL kit (Beyotime) for visualization. The signal intensity of the proteins was determined by ImageJ software. The antibodies included multidrug resistance protein 1 (MRP1; bs-24241R; 1:2000; Bioss), p-glycoprotein (MDR1; bs-0563R; 1:2000; Bioss), B-cell lymphoma-2 (Bcl-2; bs-20351R; 1:2000; Bioss), BCL2-Associated X (Bax; bs-28034R; 1:2000; Bioss), FGFR1 (bs-0230R; 1:2000; Bioss) and β-actin (bs-0061R; 1:10,000; Bioss).

### Dual-Luciferase Reporter Assay

CircARVCF-wide type (WT), circARVCF-mutant (MUT), -FGFR1 3′UTR WT and FGFR1 3′UTR MUT were designed by cloning the fragments of WT circARVCF, WT FGFR1 3′UTR, as well as related mutant (lacking miR-1205 binding sites) into pGL3 vector (Promega). Then the designed vectors and miR-NC/miR-1205 were introduced into MKN-45/DDP and AGS/DDP cells. The luciferase signal was analyzing utilizing Dual-Luciferase Reporter Assay kit (Promega) after 48 h. Renilla luciferase activity was normalized to firefly luciferase activity.

### RNA Immunoprecipitation Analysis

The Magna RIP™ reagent (Millipore, Bedford, MA, United States) was used for this analysis. In brief, MKN-45/DDP and AGS/DDP cells were lysed in RIP buffer and then cell lysates were incubated with Anti-AGO2 or Anti-IgG coupled with magnetic beads. Then the immunoprecipitated RNA was extracted and qRT-PCR was used for circARVCF and miR-1205 levels.

### Murine Xenograft Model

Beijing Vital River Laboratory Animal Technology Co., Ltd. (Beijing, China) provided the male BALB/c nude mice (5-week-old). Lentivirus vectors embracing harboring sh-NC or sh-circARVCF were constructed. Then AGS/DDP cells (1 × 10^7^) with Lenti-sh-NC or Lenti-sh-circARVCF transfection were subcutaneously injected into the flank of the mice. After 7 days, the mice were intraperitoneally administrated with 6 mg/kg of DDP (Sigma-Aldrich) or PBS every 3 days (Zhi et al., 2015). The xenograft tumors were monitored every 7 days and tumor volume (0.5 × length × width^2^) was calculated. After 28 days, the mice were euthanized and tumors were weighed and preserved at −80°C for further use. This animal experiment was permitted by the Ethics Committee of Animal Research of Huai’an First People’s Hospital.

### Immunohistochemistry Assay

The xenograft tumors were sectioned at 5 μm, fixed with 4% formalin (Sigma-Aldrich) and embedded with paraffin (Sigma-Aldrich). Next, the slides were incubated with anti-FGFR1 (bs-0230R; Bioss) overnight and secondary antibody (bs-0293G-HRP) for 1 h. After that, the signal was developed with DAB solution and counterstained with hematoxylin (Sigma-Aldrich) as previously described (Wang et al., 2019b).

### Statistical Analysis

Data analysis was conducted using GraphPad Prism 7. The experiments were repeated three times. The data were exhibited as mean ± SD. Differences were compared through Student’s *t*-test or one-way ANOVA. It was defined as significant when *p* < 0.05.

## Results

### CircARVCF Was Upregulated in DDP-Resistant GC Tissues and Cells

**FIGURE 1:**
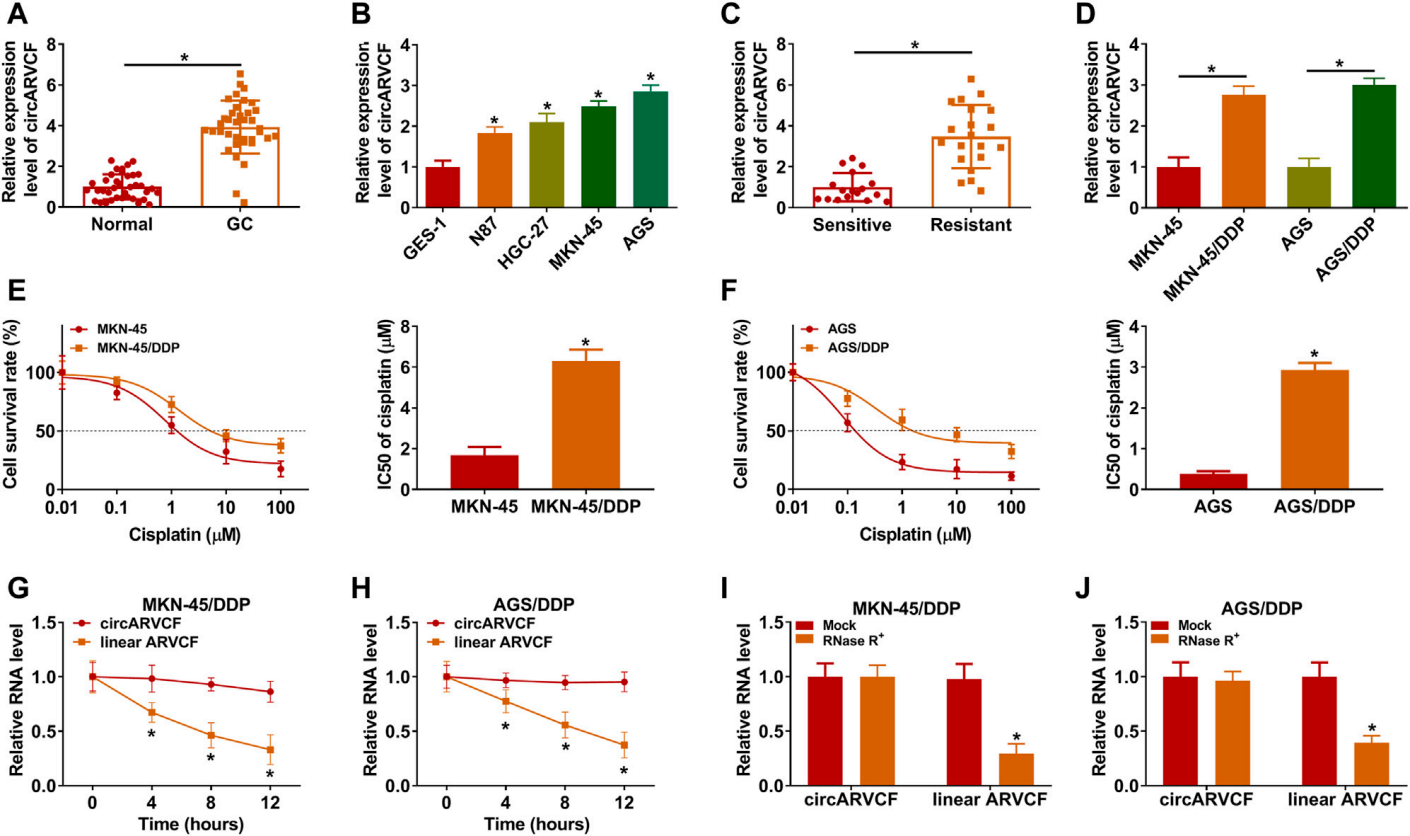
CircARVCF was overexpressed in DDP-resistant GC tissues and cells. **(A)** CircARVCF expression in GC tissues and normal tissues was detected *via* qRT-PCR. **(B)** CircARVCF expression in GES-1, N87, HGC-27, MKN-45, and AGS cells was detected by qRT-PCR. **(C)** CircARVCF expression in DDP-resistant GC tissues and DDP-sensitive GC tissues was detected by qRT-PCR. **(D)** CircARVCF expression in MKN-45, MKN-45/DDP, AGS, and AGS/DDP cells was detected by qRT-PCR. **(E,F)** IC50 of cisplatin in MKN-45, MKN-45/DDP, AGS, and AGS/DDP cells was assessed by CCK-8 assay. **(G,H)** The levels of circARVCF and ARVCF in MKN-45/DDP and AGS/DDP cells treated with Actinomycin D were examined by qRT-PCR. **(I,J)** Total RNA in MKN-45/DDP and AGS/DDP cells was treated with RNase R and then circARVCF and linear ARVCF levels were detected by qRT-PCR. *p < 0.05.

As exhibited in Figures 1A,B, circARVCF was overexpressed in GC tissues and cell lines compared to normal tissues and cell lines. To explore the functions of circARVCF in the chemoresistance of GC, the expression of circARVCF in DDP-resistant GC tissues and cells was detected by qRT-PCR. The results showed that circARVCF was upregulated in DDP-resistant GC tissues compared to DDP-sensitive GC tissues (Figure 1C). Compared to MKN-45 and AGS cells, circARVCF was increased in MKN-45/DDP and AGS/DDP cells (Figure 1D). CCK-8 assay showed that the cisplatin resistance of MKN-45/DDP and AGS/DDP cells was enhanced for the elevation of IC50 of cisplatin (Figures 1E,F). Moreover, Actinomycin D assay indicated that circARVCF possessed a longer half-life than linear ARVCF after Actinomycin D exposure (Figures 1G,H). RNase R assay indicated that circARVCF was resistant to RNase R digestion, while linear ARVCF was digested by RNase R (Figures 1I,J).

### CircARVCF Knockdown Repressed the Resistance of DDP-Resistant GC Cells to DDP

**FIGURE 2:**
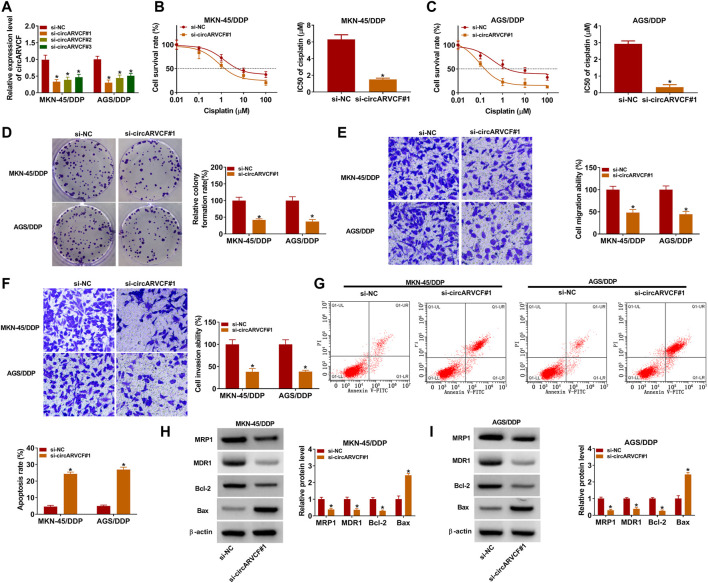
CircARVCF knockdown suppressed DDP resistance in DDP-resistant GC cells. **(A)** The expression of circARVCF in MKN-45/DDP and AGS/DDP cells transfected with si-circARVCF#1, si-circARVCF#2, si-circARVCF#3, or si-NC was detected by qRT-PCR. **(B–I)** MKN-45/DDP and AGS/DDP cells were transfected with si-NC or si-circARVCF#1. **(B,C)** IC50 of cisplatin in MKN-45/DDP and AGS/DDP cells was examined with CCK-8 assay. **(D)** The colony formation of MKN-45/DDP and AGS/DDP cells was evaluated by colony formation assay. **(E,F)** The migration and invasion of MKN-45/DDP and AGS/DDP cells were tested by transwell assay. **(G)** The apoptosis of MKN-45/DDP and AGS/DDP cells was analyzed by flow cytometry analysis. **(H,I)** The protein levels of MRP1, MDR1, Bcl-2 and Bax in MKN-45/DDP and AGS/DDP cells were measured *via* western blot assay. *p < 0.05.

To explore the exact roles of circARVCF in DDP resistance in GC, si-circARVCF#1, si-circARVCF#2, or si-circARVCF#3 was transfected into MKN-45/DDP and AGS/DDP cells to silence circARVCF expression. As presented in Figure 2A, the transfection of si-circARVCF#1, si-circARVCF#2, or si-circARVCF#3 markedly reduced circARVCF expression in MKN-45/DDP and AGS/DDP cells compared to si-NC transfected cells. Then CCK-8 assay showed that circARVCF knockdown suppressed DDP resistance in MKN-45/DDP and AGS/DDP cells compared to si-NC control groups (Figures 2B,C). Colony formation assay showed that circARVCF silencing suppressed the colony formation capacity of MKN-45/DDP and AGS/DDP cells compared to si-NC control groups (Figure 2D). The results of transwell assay indicated that circARVCF knockdown repressed the migration and invasion of MKN-45/DDP and AGS/DDP cells in comparison with si-NC control groups (Figures 2E,F). Flow cytometry analysis exhibited that circARVCF deficiency led to a marked promotion in the apoptosis of MKN-45/DDP and AGS/DDP cells (Figure 2G). In addition, our results showed that circARVCF interference decreased the levels of drug resistance-related genes (MRP1 and MDR1) and anti-apoptotic protein Bcl-2 and increased pro-apoptotic protein Bax in MKN-45/DDP and AGS/DDP cells (Figures 2H,I). Collectively, circARVCF promoted DDP resistance in DDP-resistant GC cells.

### CircARVCF Directly Targeted miR-1205 to Regulate miR-1205 Expression

**FIGURE 3:**
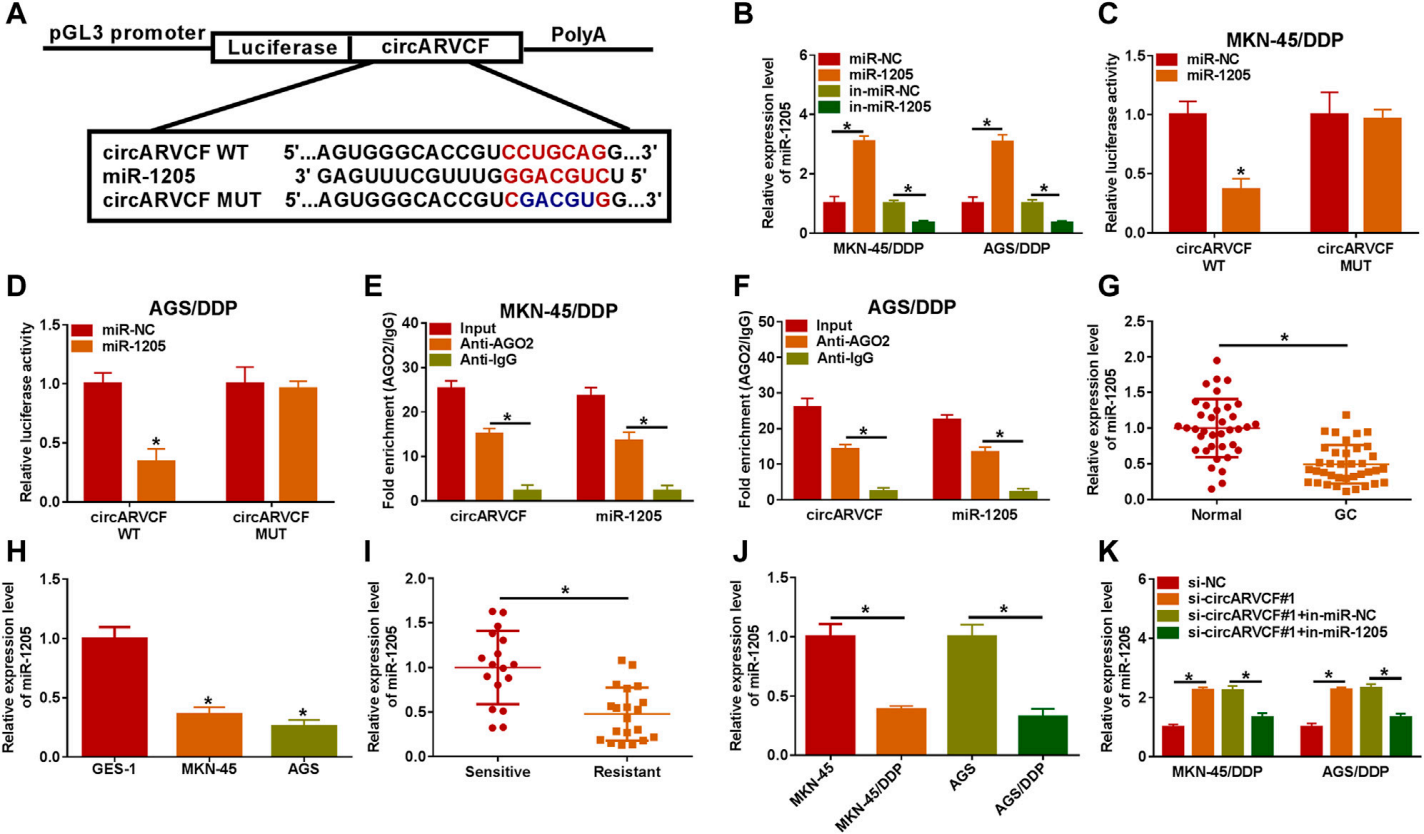
MiR-1205 was the target of circARVCF. **(A)** The complementary sequences between circARVCF and miR-1205. **(B)** The expression of miR-1205 in MKN-45/DDP and AGS/DDP cells transfected with miR-NC, miR-1205, in-miR-NC or in-miR-1205 was detected by qRT-PCR. **(C–F)** The interaction between miR-1205 and circARVCF was demonstrated by dual-luciferase reporter assay and RIP assay. **(G,H)** The expression of miR-1205 in GC tissues, normal tissues, GC cells and normal cells was detected by qRT-PCR. **(I,J)** The expression of miR-1205 in DDP-resistant GC tissues and cells was examined with qRT-PCR. **(K)** The expression of miR-1205 in MKN-45/DDP and AGS/DDP cells with si-NC, si-circARVCF#1, si-circARVCF#1+in-miR-NC, or si-circARVCF#1+in-miR-1205 was detected by qRT-PCR. *p < 0.05.

It was widely documented that circRNAs can act as the sponge for miRNAs (Zhong et al., 2018). Thus, we further explored the underlying mechanism of circARVCF in the chemoresistance of GC through searching circinteractome (https://circinteractome.nia.nih.gov/api/v2/mirnasearch?circular_rna_query=hsa_circ_0092330&mirna_query=&submit=miRNA+Target+Search) and found that miR-1205 was the target of circARVCF (Figure 3A). MiR-1205 mimic transfection apparently increased miR-1205 expression and miR-1205 inhibitor (in-miR-1205) transfection markedly reduced miR-1205 expression in MKN-45/DDP and AGS/DDP cells (Figure 3B). Then dual-luciferase reporter assay and RIP assay were performed to analyze the relationship between circARVCF and miR-1206. The results showed that miR-1205 overexpression restrained the luciferase activity of circARVCF WT in MKN-45/DDP and AGS/DDP cells, but had no effect on circARVCF MUT luciferase activity (Figures 3C,D). RIP assay showed that circARVCF and miR-1205 enrichment was increased by Anti-AGO2 RIP compared to Anti-IgG and Input control groups (Figures 3E,F). As expected, miR-1205 was lowly expressed in GC tissues and cells compared to normal tissues and cells (Figures 3G,H). Furthermore, miR-1205 was downregulated in DDP-resistant GC tissues and cells in comparison with DDP-sensitive GC tissues and cells (Figures 3I,J). In addition, it was found that circARVCF knockdown significantly increased miR-1205 expression in MKN-45/DDP and AGS/DDP cells, while in-miR-1205 transfection reversed the effect (Figure 3K). To summarize, circARVCF directly targeted miR-1205 to regulate miR-1205 expression.

### CircARVCF Knockdown Inhibited DDP Resistance in DDP-Resistant GC Cells by Targeting miR-1205

**FIGURE 4:**
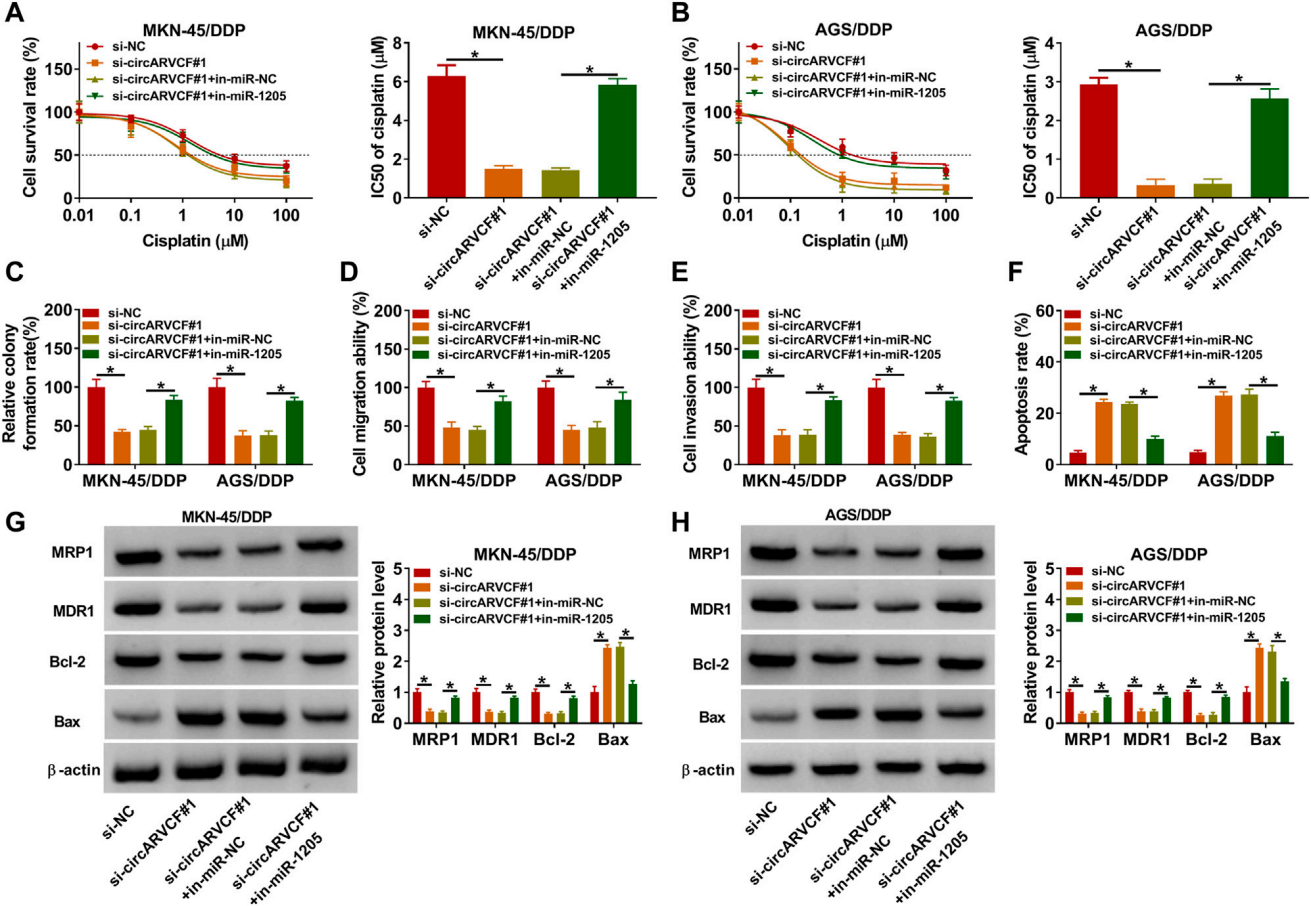
Inhibition of miR-1205 reversed the effects of circARVCF knockdown on DDP resistance, cell colony formation, migration, invasion, and apoptosis in DDP-resistant GC cells. MKN-45/DDP and AGS/DDP cells were transfected with si-NC, si-circARVCF#1, si-circARVCF#1+in-miR-NC, or si-circARVCF#1+in-miR-1205. **(A,B)** IC50 of cisplatin was estimated by CCK-8 assay. **(C)** The colony formation of MKN-45/DDP and AGS/DDP cells was examined with colony formation assay. **(D,E)** The migration and invasion of MKN-45/DDP and AGS/DDP cells were assessed by transwell assay. **(F)** The apoptosis of MKN-45/DDP and AGS/DDP cells was analyzed by flow cytometry analysis. **(G,H)** The protein levels of MRP1, MDR1, Bcl-2 and Bax in MKN-45/DDP and AGS/DDP cells were measured *via* western blot assay. *p < 0.05.

Thereafter, whether circARVCF could regulate DDP resistance by targeting miR-1205 was clarified by transfecting MKN-45/DDP and AGS/DDP cells with si-NC, si-circARVCF#1, si-circARVCF#1+in-miR-NC, or si-circARVCF#1+in-miR-1205. CCK-8 assay showed that the inhibitory effect of circARVCF knockdown on the resistance of MKN-45/DDP and AGS/DDP cells to DDP was recovered by miR-1205 inhibition (Figures 4A,B). Colony formation assay showed that circARVCF silencing repressed MKN-45/DDP and AGS/DDP cell colony formation ability, while miR-1205 inhibition reversed the effect (Figure 4C). As illustrated by transwell assay, circARVCF deficiency hampered MKN-45/DDP and AGS/DDP cells to migrate and invade, whereas these impacts were weakened by reducing miR-1205 (Figures 4D,E). Flow cytometry analysis indicated that circARVCF silencing facilitated the apoptosis of MKN-45/DDP and AGS/DDP cells, with miR-1205 inhibition abated the impact (Figure 4F). In addition, circARVCF silencing reduced the levels of MRP1, MDR1 and Bcl-2 and elevated the level of Bax in MKN-45/DDP and AGS/DDP cells, while miR-1205 downregulation reversed the effects (Figures 4G,H). These findings suggested that circARVCF inhibited DDP resistance in DDP-resistant GC cells by binding to miR-1205.

### FGFR1 Was the Target Gene of miR-1205

**FIGURE 5:**
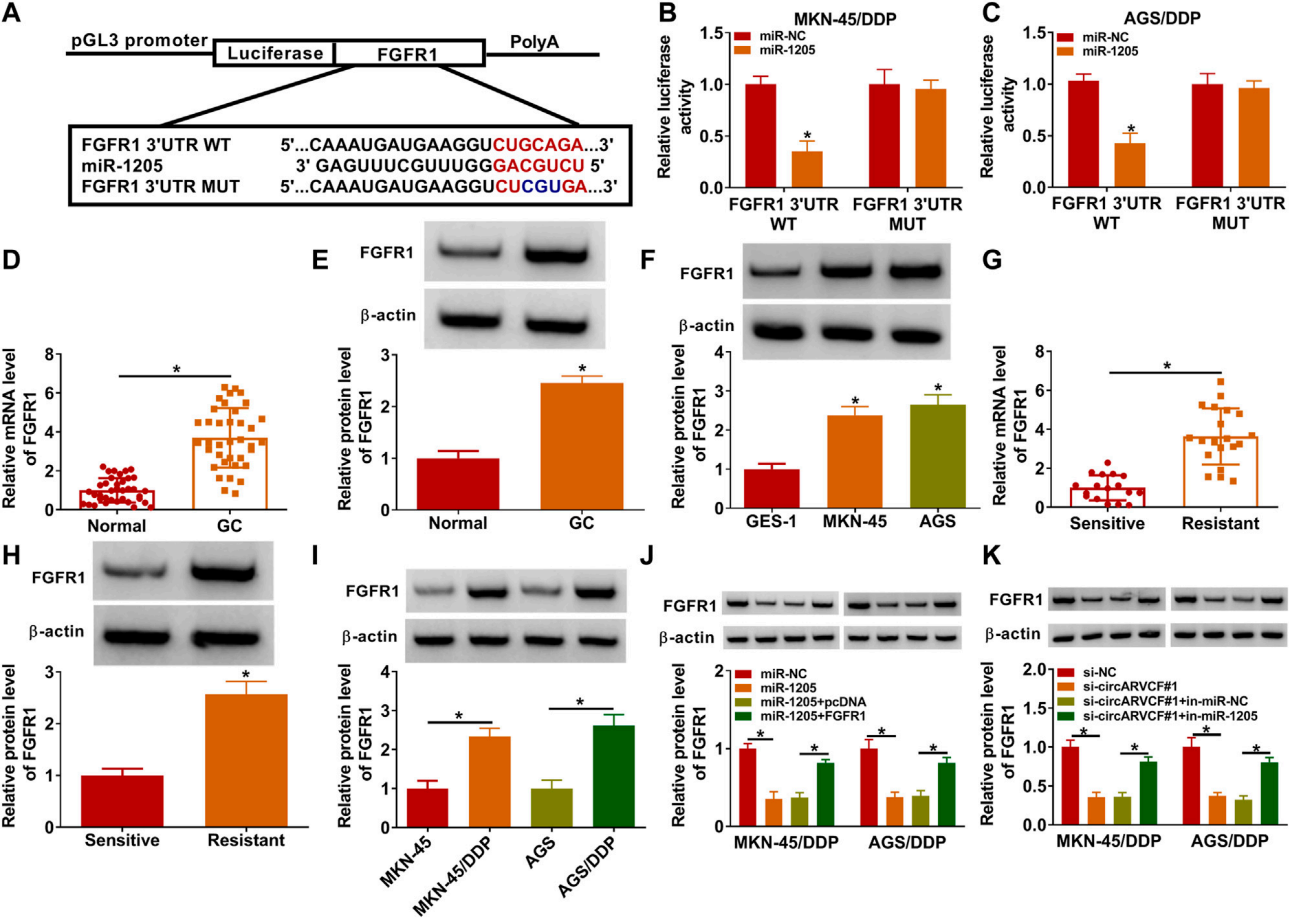
CircARVCF regulated FGFR1 expression by targeting miR-1205. **(A)** The complementary sequences between FGFR1 and miR-1205 were exhibited. **(B,C)** The relationship between FGFR1 and miR-1205 was analyzed by dual-luciferase reporter assay. **(D,E)** The mRNA and protein levels of FGFR1 in GC tissues and normal tissues were measured by qRT-PCR or western blot assay. **(F)** The protein level of FGFR1 in GES-1, MKN-45 and AGS cells was measured via western blot assay. **(G,H)** The mRNA and protein levels of FGFR1 in DDP-resistant and DDP-sensitive GC tissues were quantified by qRT-PCR or western blot. **(I)** The protein level of FGFR1 in MKN-45, MKN-45/DDP, AGS and AGS/DDP cells was measured via western blot assay. **(J)** The protein level of FGFR1 in MKN-45/DDP and AGS/DDP cells transfected with miR-NC, miR-1205, miR-1205 + pcDNA or miR-1205 + FGFR1 was measured via western blot assay. **(K)** The protein level of FGFR1 in MKN-45/DDP and AGS/DDP cells transfected with si-NC, si-circARVCF#1, si-circARVCF#1+in-miR-NC or si-circARVCF#1+in-miE-1205 was measured through western blot assay. *p < 0.05.

Subsequently, the potential target genes of miR-1205 wwere analyzed by Targetscan (http://www.targetscan.org/cgi-bin/targetscan/vert_71/view_gene.cgi?rs=ENST00000397091.5&taxid=9606&members=miR-1205&showcnc=1&shownc=1&shownc_nc=1&showncf1=1&showncf2=1&subset=1). As a result, FGFR1 was found to contain the binding sites of miR-1205 (Figure 5A). Then dual-luciferase reporter assay further demonstrated the interaction between miR-1205 and FGFR1 for the luciferase activity of FGFR1 3′UTR WT in MKN-45/DDP and AGS/DDP cells was inhibited after miR-1205 overexpression (Figures 5B,C). Indeed, the mRNA and protein levels of FGFR1 were upregulated in GC tissues compared to adjacent normal tissues (Figures 5D,E). Compared to GES-1 cells, FGFR1 protein level was increased in MKN-45 and AGS cells (Figure 5F). Moreover, FGFR1 mRNA and protein levels were increased in DDP-resistant GC tissues compared to DDP-sensitive GC tissues (Figures 5G,H). The protein level of FGFR1 was higher in MKN-45/DDP and AGS/DDP cells than MKN-45 and AGS cells (Figure 5I). Besides, miR-1205 overexpression reduced FGFR1 protein level in MKN-45/DDP and AGS/DDP cells, while the effect was rescued by elevating FGFR1 (Figure 5J). Remarkably, circARVCF interference decreased the protein level of FGFR1 in MKN-45/DDP and AGS/DDP cells, whereas miR-1205 inhibition restored the impact (Figure 5K). Taken together, circARVCF directly targeted miR-1205 to positively regulate FGFR1 expression.

### MiR-1205 Overexpression Suppressed DDP Resistance in DDP-Resistant GC Cells *via* Regulating FGFR1 Expression

**FIGURE 6:**
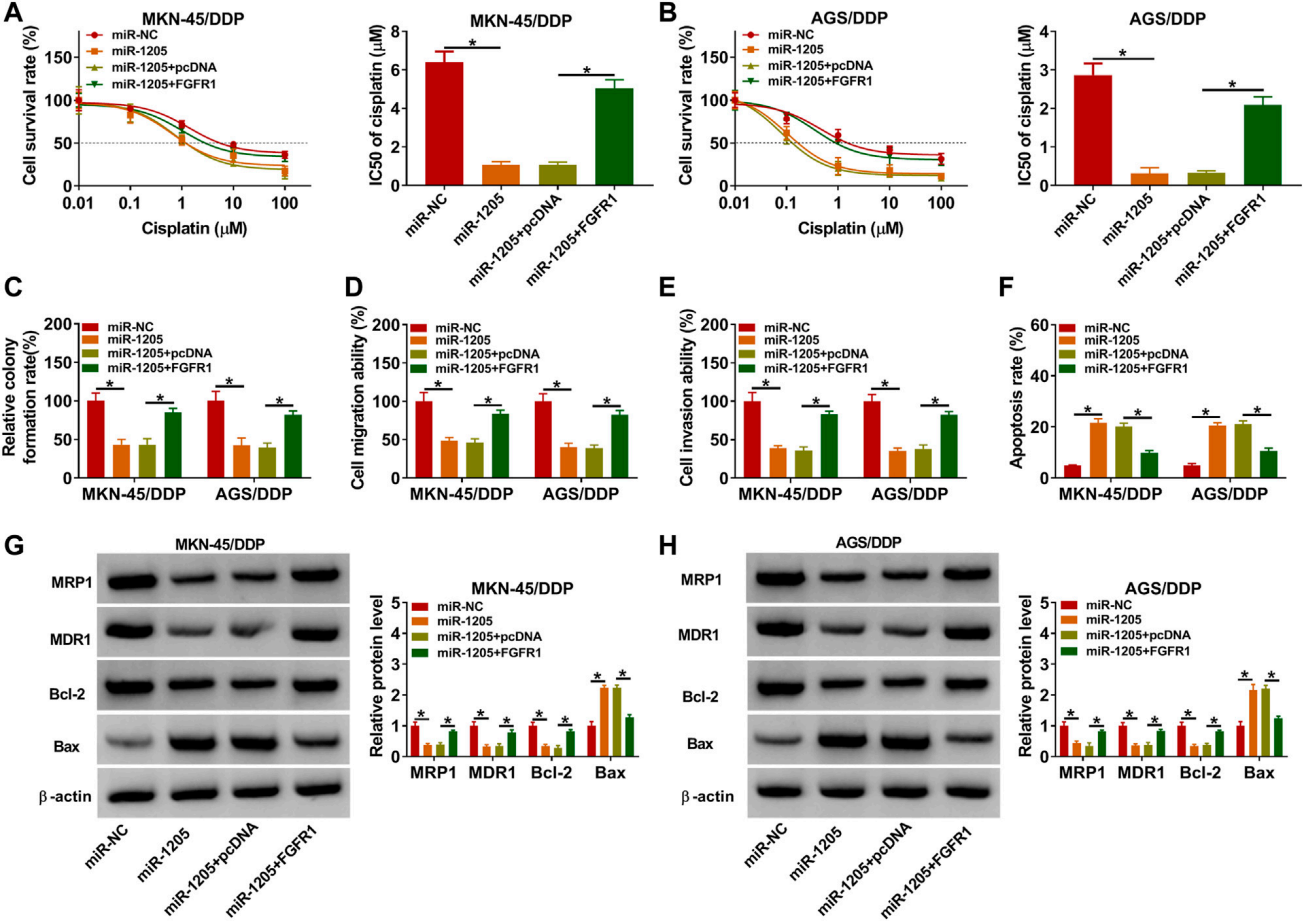
MiR-1205 regulated the DDP resistance and malignant behaviors of DDP-resistant GC cells by targeting FGFR1. MKN-45/DDP and AGS/DDP cells were transfected with miR-NC, miR-1205, miR-1205 + pcDNA or miR-1205 + FGFR1. **(A,B)** DDP resistance was analyzed by CCK-8 assay. **(C–F)** The colony formation, migration, invasion and apoptosis of MKN-45/DDP and AGS/DDP cells were evaluated by colony formation assay, transwell assay and flow cytometry analysis, respectively. **(G,H)** The protein levels of MRP1, MDR1, Bcl-2 and Bax in MKN-45/DDP and AGS/DDP cells were measured via western blot assay. *p < 0.05.

To explore whether miR-1205 could regulate the resistance of DDP-resistant GC cells to DDP, MKN-45/DDP, and AGS/DDP cells were introduced with miR-NC, miR-1205, miR-1205 + pcDNA or miR-1205 + FGFR1. As indicated by CCK-8 assay, miR-1205 overexpression inhibited DDP resistance in MKN-45/DDP and AGS/DDP cells, while FGFR1 elevation ameliorated the effect (Figures 6A,B). The results of colony formation assay, transwell assay and flow cytometry analysis exhibited that miR-1205 overexpression restrained cell colony formation, migration and invasion and promoted apoptosis in MKN-45/DDP and AGS/DDP cells, while these impacts were abrogated by increasing FGFR1 expression (Figures 6C–F). Additionally, miR-1205 overexpression reduced MRP1, MDR1 and Bcl-2 levels and elevated Bax level in MKN-45/DDP and AGS/DDP cells, with FGFR1 enhancement ameliorated the effects (Figures 6G,H). To sum up, miR-1205 overexpression inhibited the resistance of DDP-resistant GC cells to DDP through interacting with FGFR1.

### CircARVCF Knockdown Suppressed DDP Resistance *in Vivo*

**FIGURE 7:**
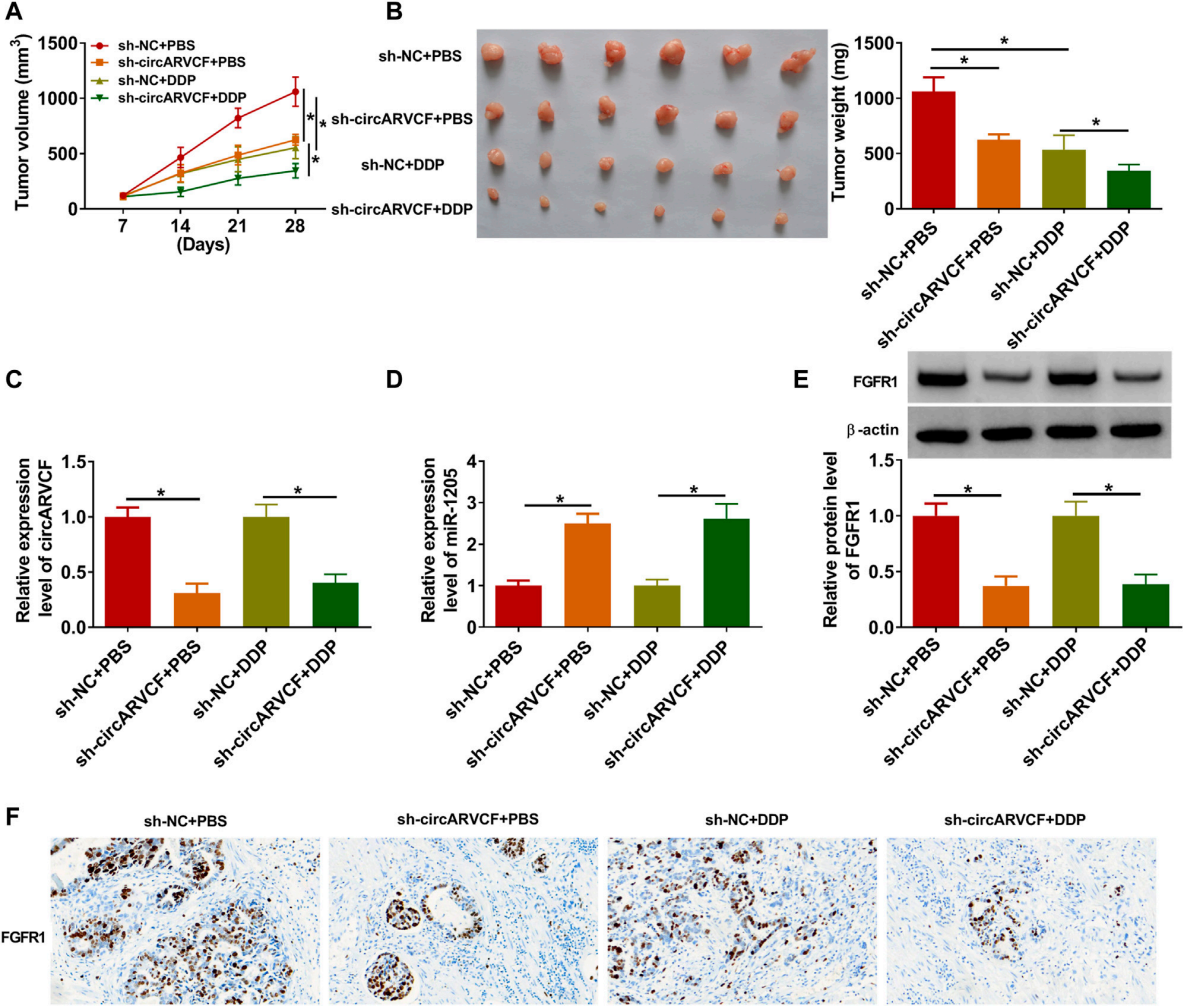
CircARVCF enhanced DDP resistance in GC *in vivo*. **(A,B)** Tumor volume and tumor weight were examined. **(C,D)** The levels of circARVCF and miR-1205 in the tumors were examined by qRT-PCR. **(E,F)** The level of FGFR1 in the tumors was examined by western blot assay and IHC assay. *p < 0.05.

Finally, the role of circARVCF in DDP resistance *in vivo* was investigated. Our results showed that circARVCF knockdown and DDP treatment repressed tumor growth (including tumor volume and tumor weight) (Figures 7A,B). As determined by qRT-PCR, western blot assay and IHC assay, the levels of circARVCF and FGFR1 were decreased and the level of miR-1205 was increased in the tumors in sh-circARVCF + PBS and sh-circARVCF + DDP groups compared to sh-NC + PBS and sh-NC + DDP groups (Figures 7C–F). These results suggested the suppressive role of circARVCF knockdown on DDP resistance in GC *in vivo*.

## Discussion

Chemoresistance is the main obstacle in the process of human cancer therapy (Marin et al., 2016). CircRNAs have been disclosed to play vital roles in tumor advancement and therapeutic resistance (Wei et al., 2020). Even so, there are still large amounts of circRNAs that have not been identified. In this study, the functions of circARVCF in DDP resistance were clarified. As a result, circARVCF inhibited the sensitivity of GC to DDP through a new regulatory axis of circARVCF/miR-1205/FGFR1.

Currently, the involvement of circRNAs and miRNAs in regulating GC chemoresistance has been reported. For example, Zhong *et al.* declared that circ_0032821 facilitated OXA resistance and malignant cell behaviors in GC partially *via* influencing miR-515-5p/SOX9 axis (Zhong et al., 2021). Zhang *et al.* demonstrated the suppressive role of circ_0026359/miR-1200/POLD4 axis in DDP sensitivity of GC (Zhang et al., 2020). Circ-PVT1 enhanced the resistance of GC cells to paclitaxel by increasing ZEB1 *via* sponging miR-124-3p (Liu et al., 2019). Circ_0110805 aggravated the cisplatin resistance of GC by sponging miR-299-3p and elevating ENDOPDI (Yang et al., 2020a). CircAKT3/miR-198/PIK3RI played a promotional effect on cisplatin resistance of GC (Huang et al., 2019). These reports all indicated that chemoresistance could be modulated by circRNAs. In the present study, circARVCF was increased in DDP-resistant GC tissues and cells. Next, we performed experiments to explore the functions of circARVCF in DDP resistance of GC. It was found that circARVCF silencing inhibited IC50 of DDP, cell colony formation and metastasis and triggered apoptosis in DDP-resistant GC cells. Moreover, the *in vivo* experiments demonstrated the promotional effect of circARVCF knockdown on DDP sensitivity *in vivo*. These findings suggested that circARVCF boosted the resistance of GC to DDP.

CircRNAs can be involved in the chemoresistance of cancers by binding to miRNAs and then alter miRNA target gene expression (Hansen et al., 2013). Herein, circARVCF could interact with miR-1205, which then targeted FGFR1 to alter FGFR1 expression. MiR-1205 has been reported to repress the metastasis and proliferation of GC cells *via* circCYFIP2/miR-1205/E2F1 pathway (Lin et al., 2020). Moreover, miR-1205 was associated with sorafenib resistance in hepatocellular carcinoma by interacting with circFN1 and E2F1 (Yang et al., 2020b). Nonetheless, we firstly explored the functions of miR-1205 in DDP sensitivity of GC. It was found that miR-1205 was reduced in DDP-resistant GC. Inhibition of miR-1205 restored circARVCF knockdown-mediated inhibitory influence on DDP sensitivity of chemo-resistant GC cells. Overexpression of miR-1205 enhanced DDP sensitivity and restrained malignant cell phenotypes in DDP-resistant GC cells.

FGFR1 can be activated to influence the proliferation, survival, migration and differentiation of tumor cells (Neal et al., 1985; Guo et al., 2017). FGFR1 could serve as the target for diverse miRNAs, such as miR-198 (Gu et al., 2019), miR-497 (Xie et al., 2018), and miR-133b (Wen et al., 2013). However, FGFR1 was verified to be the target gene of miR-1205 for the first time. Moreover, FGFR1 participated in the regulation of chemosensitivity in several cancers (Hong et al., 2020; Kong et al., 2020). In the research, FGFR1 could combine with miR-1205 to alter DDP resistance in GC. In addtion, FGFR1 has been reported to regulate the biologocal processess, including wound repair, tissue regeneration, inflammation and angiogenesis, which are implcated in cancer progression and drug resistance (Liang et al., 2021; Yang et al., 2021). However, whether circARVCF/miR-1205/FGFR1 axis is involved in these biological processes to regulate DDP resistance is still unclear.

**FIGURE 8:**
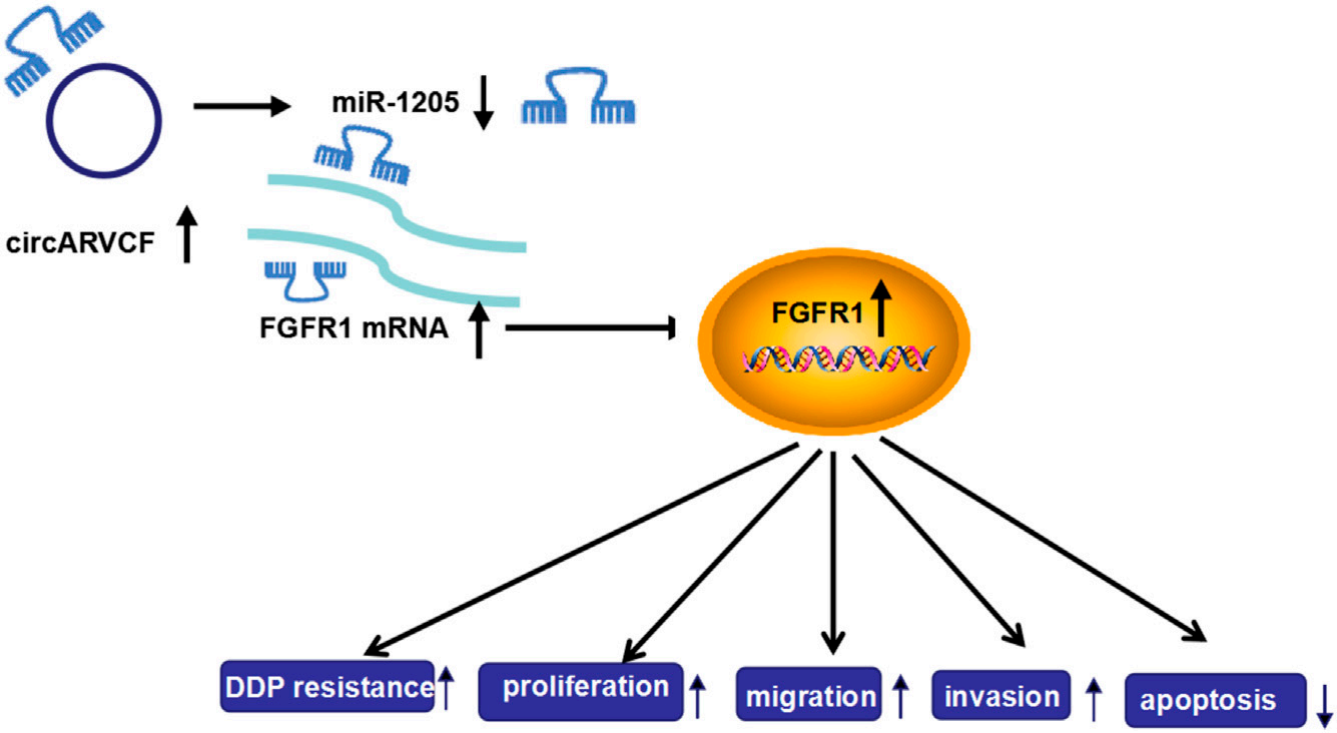
The frame diagram of circARVCF/miR-1205/FGFR1 axis in regulating DDP resistance and cell progression in GC.

Taken together, circARVCF contributed to DDP resistance and promoted tumor cell proliferation, migration, and invasion in GC by regulating miR-1205 and FGFR1 expression (Figure 8). This study deepened our understanding of the progression of chemoresistance and provided a novel insight to resist drug resistance in GC.

## Data Availability

The original contributions presented in the study are included in the article/Supplementary Material, further inquiries can be directed to the corresponding authors.
